# Lung Nodules and Hypoxemia: Any Association?

**DOI:** 10.7759/cureus.13880

**Published:** 2021-03-14

**Authors:** Konstantina Chadia, Paschalis Ntolios, Stavros Anevlavis, Paschalis Steiropoulos

**Affiliations:** 1 Pulmonology, University Hospital of Alexandroupolis, Alexandroupolis, GRC

**Keywords:** pulmonary arteriovenous malformation, interventional radiology guided embolization

## Abstract

Pulmonary arteriovenous malformations (PAVMs) consist of aberrant circulation between pulmonary arteries and veins causing right-to-left shunt, uncommon and asymptomatic in the general population. We presented two patients, one presented with unexplained dyspnea and disease limited to the lung and the other with neurologic signs and systematic disease. Both patients were diagnosed with arteriovenous malformations and received embolization treatment successfully. Both patients received embolization treatment successfully.

## Introduction

Pulmonary arteriovenous malformations (PAVMs), also known as pulmonary arteriovenous fistulae, pulmonary arteriovenous aneurysms, cavernous angiomas of the lung and pulmonary telangiectasias consist of aberrant circulation between pulmonary arteries and veins [1]. The normal anatomic barrier that should separate the pulmonary arterial from the venous circulation is lost, resulting in anatomic right-to-left shunt [2]. These lesions are uncommon in the general population with a prevalence of 1 in 2600 individuals and are 1.5-2 times more common among women than men [3-4], and usually become apparent after puberty, between the fourth and sixth decades. They can be picked up incidentally on chest imaging but also present as unexplained dyspnea or hypoxemia [2].

We hereby describe the diagnostic approach and treatment considerations in two patients. The first patient presented with unexplained dyspnea and disease limited to the lung, while the second patient with neurologic signs and systematic disease.

## Case presentation

Case 1

A 74-year-old, nonsmoker, female patient was referred to our outpatient clinic for low oxygen saturation and central cyanosis. She reported progressively worsening dyspnea (mMRC II/IV) during the last four years and nonproductive cough. Her medical history included dyslipidemia and arterial hypertension. No other symptoms were reported.

On physical examination, her pulse rate was 65 beats/min and respiratory rate 16 breaths/min. Blood pressure was 120/80 mmHg and oxygen saturation on room air was 89%. Lung and heart auscultation were normal. No murmurs or clubbing was observed. Rest of physical examination was unremarkable. Hemoglobin was 15.2 g/dL and white blood cells and differential counts were normal. Serum urea was 27 mg/dL and creatinine was 0.7 mg/dL. Rest of laboratory tests were normal. Arterial blood gases (ABGs) sampling revealed type I respiratory failure (pO2 55 mmHg, pCO2 36 mmHg, pH: 7.45, HCO3: 25 mmol/L, FiO2: 21%). Supplemental oxygen with a nasal cannula (4 L/min) provided only partial improvement of ABGs (pO2 59 mmHg, pCO2 38 mmHg, pH 7.43, HCO3: 25 mmol/L, FiO2: 33%). A chest radiograph showed a right lung nodule without other pathologic signs.

**Figure 1:**
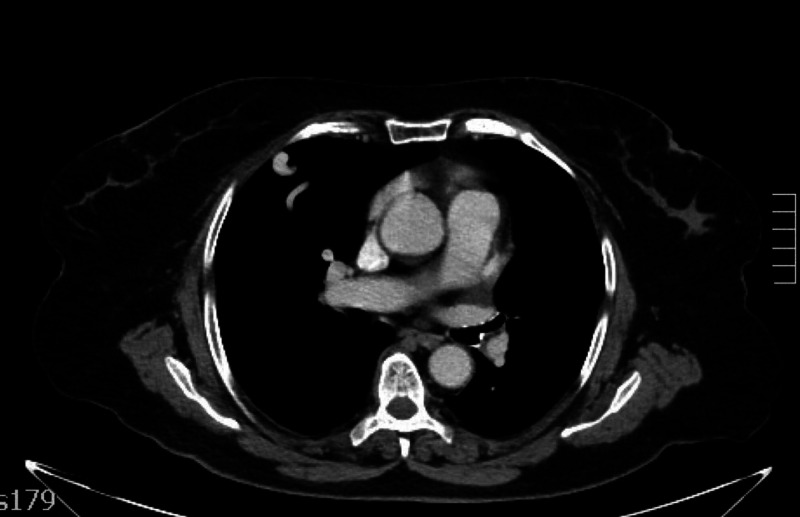
Contrast chest CT axial images shows a PAVM as a plexiform mass of dilated vascular channels. PAVM, pulmonary arteriovenous malformation

Transthoracic bubble echocardiography confirmed the presence of right-to-left shunt, without visualization of any cardiac or large vessels defects. A post IV contrast chest CT (Figure 1) revealed a 9 mm, well-defined nodule without calcification, confirming the presence of an arteriovenous malformation. Brain and abdominal CT were normal. 

Hereditary hemorrhagic telangiectasia (HHT) was excluded as a possible cause but no other cause could be identified. The patient underwent embolization by a chest radiologist with curative results.

Case 2

A 15-year-old male patient was referred to the ED, due to high fever (39°C) lasting 72 hours, cervical stiffness and frontal headache. No other symptoms were reported.

On physical examination, pulse rate was 100 beats/min, respiratory rate was 12 breaths/min, and blood pressure was 120/80 mmHg. Lung auscultation was normal. Cardiac sounds were normal with no murmurs. He did not have clubbing. His oxygen saturation was lower when supine (platypnea). Rest of physical examination was unremarkable. ABGs analysis revealed hypoxemia (pH: 7.45, pCO2: 36 mmHg, pO2: 68 mmHg, HCO3: 25 mmol/L, FiO2: 21%). Routine laboratory investigations were normal. Hemoglobin was 11.1 g/dL. Liver and renal panels as well as inflammatory markers were within normal limits. The patient was suspected to have meningitis and was admitted to the Special Infections Unit. A brain CT revealed an enlargement of the left temporal horn. Lumbar puncture was performed and results from the cerebrospinal fluid analysis were 300 red blood cells/mm3, 5,800 nuclear cells/mm3, 30% lymphocytes, and 70% polymorphonuclear cells. Glucose was 41 mg/dL, lactate dehydrogenase (LDH) 34 U/L, and cerebrospinal fluid albumin 108 g/dL. Cerebrospinal fluid analysis revealed normal cell count and low protein levels. In addition, glucose was 101 mg/dL (serum concentration was 115 mg/dL) and polymerase chain reaction (PCR), Gram stain and culture were negative for bacteria or bacterial and/or viral DNA and/or RNA. Chest radiograph was noticeable for three nodules in the right lung.

**Figure 2:**
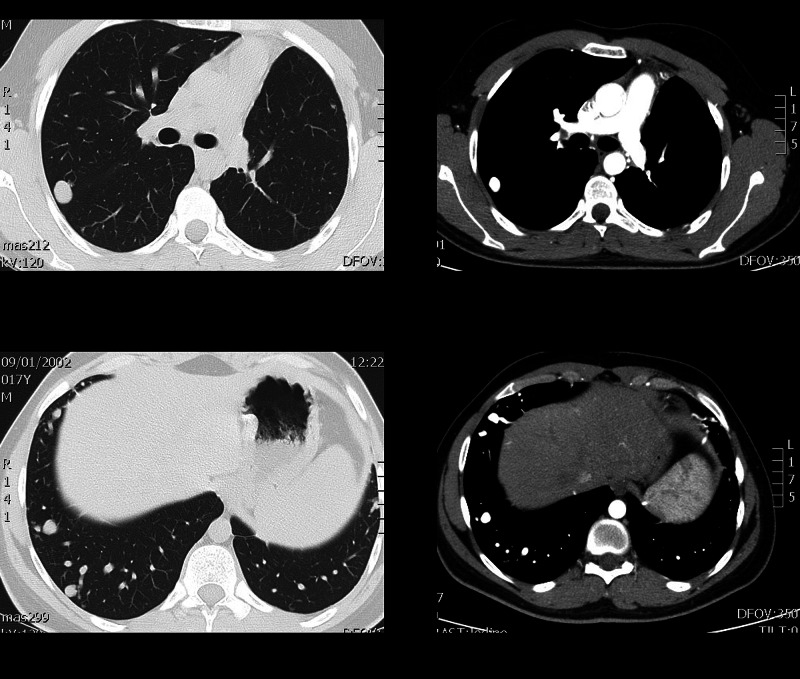
Axial CT images in the lung and soft tissue window show a large PAVM with a proximal tail-like extension and many smaller arteriovenous malformations. PAVM, pulmonary arteriovenous malformation

A subsequently post-IV contrast CT revealed numerous (>15) PAVMs in both lungs with the largest located in the right middle lobe and the superior segmental of the right lower lobe. They were perfused by branches of the pulmonary artery and had large draining veins (diameter~20 mm) (Figure 2).

**Figure 3:**
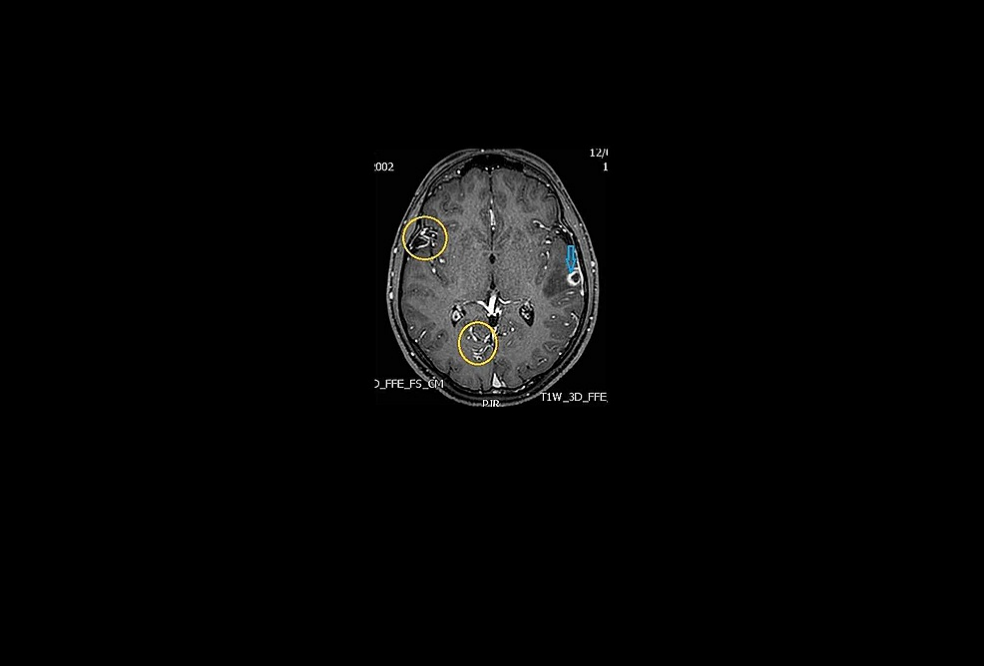
Axial images of T1 weighted MRI of brain arterionenous malformations (yellow circles). The blue arrow shows the brain abscess’s area.

A brain MRI was consistent with the diagnosis of a brain abscess with peripheral edema and hemorrhage in the right temporal lobe (Figure 3).

The largest one (12 mm) was located beside the interhemispheric fissure and was radiologically similar to those of a cavernous hemangioma. In addition, a more detailed history confirmed episodes of spontaneous nose bleeding and a first-degree relative with HHT (two out of three criteria for the diagnosis of HHT).

A contrast-enhanced pulmonary and abdominal aorta angiography was performed which confirmed the imaging findings of chest CT. Due to the presence of multiple arteriovenous malformations in multiple systems, this patient was referred to a specialized center for embolization.

## Discussion

Heredity is an important factor in arteriovenous malformations. Most PAVMs are associated with HHT (also called Rendu-Osler-Weber syndrome), an autosomal dominant vascular disorder with an estimated prevalence of 25%-98% among patients with PAVMs [5]. Individuals with HHT usually develop symptoms progressively during their lifetime [4]. The diagnosis can be established through the use of the Curaçao diagnostic criteria that include spontaneous and recurrent epistaxis, multiple mucocutaneous telangiectasia at common sites (especially lips, fingers, oral cavity, and nose), visceral involvement (gastrointestinal, pulmonary, hepatic, cerebral, spinal) and a first-degree-relative with the disease. The diagnosis can be "definite" (3+ criteria), "suspected" (2 criteria), and "unlikely" (1 criterion) [6].

Even though most arteriovenous malformations are congenital, they can be caused by a variety of acquired medical conditions, hepatic cirrhosis being the most common one [7]. Less common associations include chest trauma, mitral stenosis, actinomycosis, and Fanconi’s syndrome [8]. Surgical interventions for cyanotic congenital heart disease can also lead to PAVMs if the lung receives diminished hepatic venous return [9].

The PAVMs are classified as either simple (80%-95%) or as complex [10]. Simple PAVMs are perfused by a single feeding subsegmental artery and drained by a single vein, whereas complex PAVMs can have multiple perfusing and/or draining vessels [10]. In most situations, they are drained into the left atrium. They usually are multiple and vary in their number and distribution. Size is usually 1-5 mm but occasionally exceeding 10 mm [11]. The malformations usually manifest as a large single sac but can also appear as a lung mass consisting of dilated vessels [12].

Presentation can be asymptomatic and incidental, or with symptoms and even severe complications, some of which could be life threatening. This is usually determined by the right-to-left shunt size and the location of the malformations [9]. In some cases, brain abscess can be a complication of a bleeding brain arteriovenous malformation [13]. Spontaneous epistaxis caused by bleeding from mucosal telangiectases is the most common complaint in symptomatic patients (29%-79%), platypnea and orthodeoxia being the second (13%-56%) [14]. Symptoms and possible complications include hemoptysis (7%-30%), telangiectasis (34%-79%), bruit (29%-67%), and cyanosis (0-54%). Neurologic complications of PAVMs due to paradoxical embolization include migraines, ischemic strokes, and cerebral abscesses [2].

The diagnostic approach of PAVMs begins with the confirmation of a right-to-left shunt by transthoracic contrast echocardiography (TTCE), also known as “bubble echocardiography” [15]. TTCE has high sensitivity (100%) and low specificity (49%), a positive predictive value of 32%, and a negative predictive value of 100% [15]. Confirmation of right-to-left shunt through TTCE should be followed by a chest CT scan [15]. Treatment used to depend on the size of the feeding artery. A diameter of more than 2-3 mm was considered an indication for pulmonary angiography and embolization [9]. This threshold is no longer considered valid and all visible PAVMs are candidates for embolization [14]. Asymptomatic patients should also be subjected to treatment, due to increased risk of paradoxical emboli and hemorrhage and additionally to improve the right-to-left shunt [16]. Surgical removal is limited to cases unsuitable for embolization [9]. Patients who do not receive treatment are followed up by chest CT every three to five years [9].

## Conclusions

Pulmonary arteriovenous malformations should be part of the differential diagnosis of patients with unexplained dyspnea or hypoxemia. Initial presentation with brain abscess is rare but has been reported as a complication to a bleeding cavernous hemangioma. The presence of concomitant respiratory signs or symptoms should raise suspicion. Further diagnostic approach is important to confirm the presence of right-to-left shunt and provide an accurate visualization of PAVMs through chest CT scan. Embolization of the feeding artery remains the treatment of choice for all PAVMs regardless of size, due to the serious complications that include ischemic stroke and brain abscess in untreated patients.
